# Hearing Therapy Improves Tinnitus-Related Distress in Mildly Distressed Patients with Chronic Tinnitus and Mild-to-Moderate Hearing Loss: A Randomized-Controlled Cross-Over Design

**DOI:** 10.3390/jcm11071764

**Published:** 2022-03-22

**Authors:** Benjamin Boecking, Leonie Rausch, Stamatina Psatha, Amarjargal Nyamaa, Juliane Dettling-Papargyris, Christine Funk, Petra Brueggemann, Matthias Rose, Birgit Mazurek

**Affiliations:** 1 Tinnitus Centre, Charité—Universitätsmedizin Berlin, 10117 Berlin, Germany; benjamin.boecking@charite.de (B.B.); leonie.rausch@charite.de (L.R.); stamatina.psatha@charite.de (S.P.); nyamaa.amarjargal@charite.de (A.N.); petra.brueggemann@charite.de (P.B.); 2 Terzo Institute, ISMA AG, 96515 Sonneberg, Germany; j.dettling@terzo-institut.de (J.D.-P.); c.funk@terzo-institut.de (C.F.); 3 Medical Department, Division of Psychosomatic Medicine, Charité—Universitätsmedizin Berlin, 10117 Berlin, Germany; matthias.rose@charite.de

**Keywords:** auditory training, hearing aids, mild-to-moderate hearing loss, tinnitus-related distress, psychological epiphenomena

## Abstract

Background: The psychological effects of hearing aids and auditory training are underinvestigated. Objective: To assess the short- and long-term effects of an industry-developed auditory training on tinnitus-related distress, perceived stress, and psychological epiphenomena in patients with chronic tinnitus and mild-to-moderate hearing loss. Method: One-hundred-seventy-seven gender-stratified patients were randomized to an immediate [IIG] or delayed [DIG] intervention group. Following binaural hearing aid fitting, participants completed a CD-enhanced 14-days self-study program. Applying a randomized-controlled cross-over design, psychological measures were obtained at four times: pre-treatment/wait [IIG: t_1_; DIG: wait], post-treatment/pre-treatment [IIG: t_2_; DIG: t_1_], follow-up/post-treatment [IIG: t_3_; DIG: t_2_], and *follow-up* [DIG: t_3_]. Between- and within-group analyses investigated treatment-related effects and their stability at a 70-day follow-up. Results: Overall, distress symptom severity was mild. Unlike the DIG, the IIG showed significant improvements in tinnitus-related distress. Some psychological epiphenomena, notably anxiety, slightly improved in both groups. Within-group analyses demonstrated the stability of the tinnitus-distress-related effects, alongside uncontrolled improvements of perceived stress and mood-related symptoms at follow-up. Conclusions: The investigated hearing therapy lastingly improves tinnitus-related distress in mildly distressed patients with chronic tinnitus and mild-to-moderate hearing loss. Beneficial psychological knock-on effects deserve further investigation.

## 1. Introduction

Chronic tinnitus denotes the longstanding (>3 months) perception of sound without external acoustic stimulation [1]. Tinnitus is a common phenomenon with an estimated prevalence of 10–15% in the adult population [2]; however, prevalence rates differ widely between studies [3]. Whilst a majority of people adjust to the percept and report little emotional distress [4], some people experience considerable psychological distress in its wake [5,6]. Such tinnitus-related distress is commonly measured using self-report questionnaires, amongst which the Tinnitus Questionnaire (TQ), Tinnitus Handicap Inventory (THI), and Tinnitus Functional Index (TFI) are the most common. These measures’ total scores suitably capture both tinnitus-related distress and intervention-related change [7]; however, some content differences remain with the TQ and THI, emphasizing psychological over more audiological or functional impairments, respectively (TFI).

Whilst chronic tinnitus likely develops at the interface of various psychobiological influences [1], hearing loss has been identified as one of its main risk factors [2,8]. It has been found to correlate with tinnitus loudness [9], tinnitus-related distress [10], and—somewhat unsurprisingly—poorer speech-comprehension-in-noise [11,12,13]—which is further commonly impaired in tinnitus patients [14]. In order to compensate for hearing loss-related difficulties, hearing aids can offer an important interventional strategy [15]. Hearing aids have been found to improve individuals’ hearing ability, as measured by self-report [16] or audiological data (e.g., speech-reception threshold in noise [17]). Further, hearing aids appear to ameliorate some forms of chronic tinnitus [18,19,20,21]—potentially through reducing tinnitus awareness via an enhanced perception of external auditory input [22] or, for some patients, through psychological processes such as enhanced experiences of self-efficacy, control, or social inclusion.

Psychologically, chronic tinnitus, hearing loss, and difficulties with speech-comprehension-in-noise hold the potential to negatively affect individuals’ well-being—notably by causing or exacerbating anxiety, low mood, or perceived stress [14,23,24,25,26,27,28]. Therefore, hearing aids can be regarded as one important tool for the alleviation of hearing difficulties and—secondarily—tinnitus-related or broader psychological distress [15,29]. However, although there are several studies [30,31,32] that examine the effect of hearing aids and auditory training on hearing-related outcomes (such as speech-comprehension-in-noise or sound localization), potential effects of such an intervention on tinnitus-related distress and psychological parameters remain underinvestigated.

The present study aims to fill this gap and applies a randomized, controlled, three-timepoint cross-over design to investigate the effects of an industry-developed hearing therapy (which combines binaural hearing aid fitting with a specifically developed auditory training) on tinnitus-related distress, perceived stress, and psychological epiphenomena in patients with chronic tinnitus and mild-to-moderate hearing loss. We hypothesized:

1. Compared to a waiting, delayed-intervention group (DIG), an immediate intervention group (IIG) shows higher reductions in tinnitus-related distress and psychological distress following Terzo© (Sonneberg, Germany) hearing therapy;
2. Given the primarily audiological–cognitive focus of the intervention, treatment-related change may be most strongly reflected in TFI (vs. TQ or THI) scores;
3. Any observed effects will be stable at a 70-day follow-up.

## 2. Materials and Methods

### 2.1. Participants

Between 2018 and 2020, 177 participants (54.2% female; *M*_age_ = 59.61, *SD* = 7.46) were recruited from Charité Universitätsmedizin, Berlin. Adults (aged ≥ 18 years) with a tinnitus duration of more than 3 months and mild-to-moderate hearing loss were included in the study (averaged pure tone audiometry [PTA] thresholds (in decibel [dB]: *M*_right_ = 37.94, *SD* = 8.54; *M*_left_ = 35.97, *SD* = 8.43; classification: <20 = normal; 20–40 = mild; 41–60 = moderate; >60 = severe [33]). Past psychotherapeutic treatment, present psychiatric treatment, and current hearing aid use were recorded. Patients with past or present diagnoses of Ménière’s disease, acoustic neuroma or other tumours (e.g., brain tumour), identifiable organic causes of tinnitus (e.g., epilepsy), severe psychological conditions requiring treatment in their own right, severe hearing impairment, indication for a cochlea implant, ototoxic medication (e.g., diuretics), drug, alcohol or medication addiction, chemotherapy, or insufficient mastery of the German language were excluded from study participation. In order to detect an improvement of seven (*SD* = 15) points on the Tinnitus Questionnaire with an assumed alpha of 0.05 and a power of 0.80, *n* = 58 subjects were required per arm. Assuming an average drop-out rate of 20% (=12 additional subjects), seventy subjects were necessary. In the present study, the target *n* was eventually set to 75 treatment completers per arm.

See Table 1 for an overview of patient characteristics.

### 2.2. Procedure

Participants were informed about the scope and aims of the study and signed an informed consent agreement. The Charité Universitätsmedizin’s ethics committee approved the study (EA1/114/17). All methods were carried out in accordance with relevant guidelines and regulations. The original protocol was retrospectively registered with the DRKS (“Deutsches Register für Klinische Studien”—German Registry of Clinical Studies; registration number DRKS00015312; retrospectively registered on 17 September 2021), which meets the International Committee for Medical Journal Editors’ (ICMJE) clinical trial registration requirements (https://www.drks.de/drks_web/navigate.do?navigationId=trial.HTML&TRIAL_ID=DRKS00015312, accessed on 9 August 2021).

For the randomization protocol, two types of identical envelopes were created. The envelopes contained a card with a printed “A” (IIG; 75) or “B” (DIG; 75) stamp, respectively. Following a simple randomization protocol, an enrolling clinician (NA) presented participants with a box from which an envelope was drawn. The enrolling clinician was blind to the drawn intervention. Following this allocation procedure, participants met with a Terzo employee, handed over the envelope, and the respective trial protocol and procedures were explained. Randomization was continued until the target *n* of 75 patients was reached in both treatment arms. The present study reports all available data from all participants that were included in the initial randomization process (*n* = 177).

### 2.3. Study Protocol

**Figure 1:**
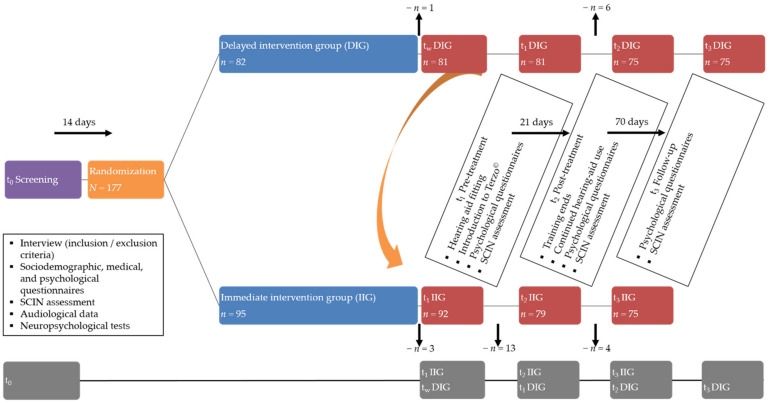
Overview of the randomized-controlled cross-over design, measurement timepoints, and dropouts. IIG = immediate intervention group and DIG = delayed intervention group. SCIN = Speech-comprehension-in-noise; t_1_ = Pre-treatment; t_2_ = Post-treatment; t_3_ = Follow-up; and t_w_ = Waiting timepoint (DIG only). Dropout rates are indicated for each arm and measurement timepoint.

**Figure 2:**
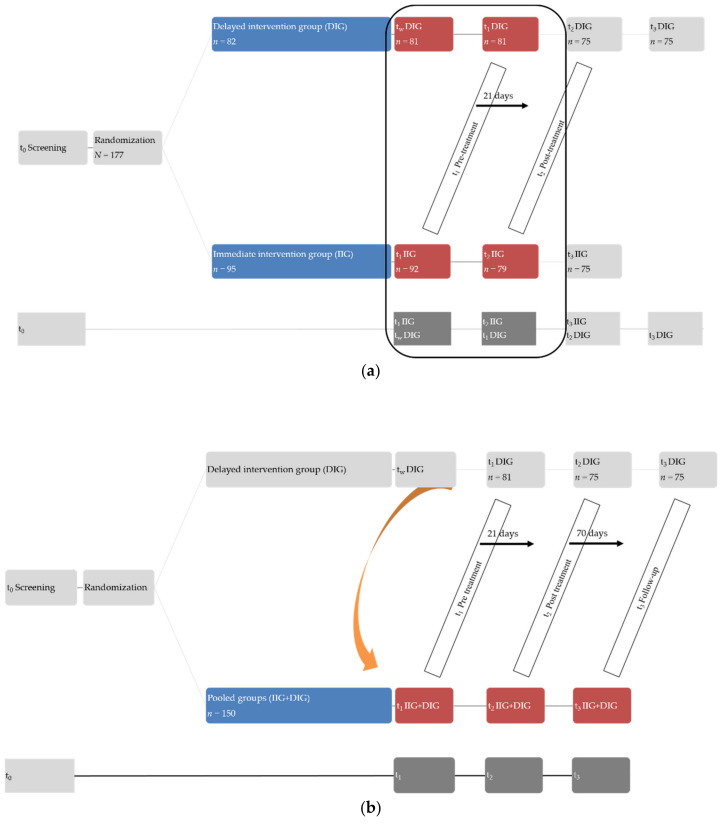
Between-subject analyses (**a**) and within-subject analyses (**b**), investigating treatment-related effects. Note: the IIG and DIG did not differ on any of the investigated outcome measures at pre-treatment (t_1_IIG; t_1_DIG).

Following recruitment and prior to treatment onset, participants completed an initial data screening, the results of which are reported elsewhere [34]. By applying a randomized-controlled cross-over design, participants were then randomized to an immediate or delayed intervention group. Tinnitus-related distress, perceived stress, and psychological epiphenomena were obtained at four times: pre-treatment/wait [IIG: t_1_; DIG: wait], post-treatment/pre-treatment [IIG: t_2_; DIG: t_1_], follow-up/post-treatment [IIG: t_3_; DIG: t_2_], and follow-up [DIG: t_3_] (Figure 1). Between and within-group analyses investigated treatment-related psychological effects and their stability over a 70-day follow-up period after the end of the intervention phase (Figure 2).

### 2.4. Hearing Ability

Prior to study inclusion, participants’ hearing ability was tested using pure tone audiometry (PTA). Here, participants indicated the quietest detectable sound (dB) at several points across the frequency range (250 Hertz [Hz], 500 Hz, 1000 Hz, 1500 Hz, 2000 Hz, 3000 Hz, 4000 Hz, 6000 Hz, 8000 Hz, and 10,000 Hz). Right-sided tinnitus perception was reported by 15 participants (8.5%); left-sided by 31 participants (17.5%), and bilateral by 131 participants (74%). Pure (sinus) tone perception was reported by 121 participants (68.4%) and narrow-band noise by 54 participants (30.5%). The average reported tinnitus frequency was 5.98 kilohertz (kHz; *SD* = 2.46) at 55.91 dB (*SD* = 19.24).

### 2.5. Terzo© Hearing Therapy

Terzo© hearing therapy [35] was originally developed for patients with hearing loss. It is characterized by a combination of hearing aid fitting, and a Terzo-specific auditory training.

During Terzo© hearing therapy, subjects were initially provided with brief educational counselling about (1) tinnitus, (2) the mechanisms of tinnitus onset and the relationship between tinnitus and hearing loss, as well as (3) principles and methods of treatment with hearing aids. In addition, subjects were prepared for their acoustic impressions when wearing hearing aids in order to improve the acceptance of hearing aid amplification. All participants underwent binaural hearing aid fitting and, subsequently, completed the auditory training. Hearing aid fittings were adjusted to subjects’ individual levels of hearing loss. Because the present sample included participants with mild-to-moderate hearing loss (i.e., of <30 dB), fittings were based on the Desired Sensation Level (DSL), v5 child’s formula to maximize speech audibility across a variety of real-world settings [36,37]. Ear moulds were used routinely, and language-specific adaptive parameters were largely deactivated.

Following a one-week adjustment period, participants were instructed to independently practice a standardized training intervention for approximately 1 h/day over a period of 14 days. The training intervention comprised a combination of auditory materials (CD) and daily, workbook-based exercises aimed to improve speech-comprehension-in-noise. The exercises included comprehension tasks pertaining to numbers, texts, similar-sounding words, syllables, and required mnestic as well as concentration elements. Thematic blocks were labelled (1) speech comprehension with and without noise, (2) concentration, (3) acoustic retention, (4) semantic memory, and (5) acoustic crossword puzzles. The training manual featured a variety of sequential exercises that were linked to specific days of the intervention period. Participants could record their progress in daily protocol sheets.

The present paper reports the effects of Terzo© hearing therapy on tinnitus-related distress and psychological wellbeing. Training intensity was quantified using participants’ self-reports obtained at the post-treatment timepoint as well as the hearing aids’ automated usage recordings. At the end of the intervention (post-treatment), participants returned the training CD. Hearing aids and self-instruction materials were returned at follow-up.

### 2.6. Measures

#### 2.6.1. Tinnitus-Related Distress

The German version of the Tinnitus Questionnaire (TQ) by Goebel and Hiller [38] assesses tinnitus-related distress. It is widely established in Germany and features 52 items that are answered on a 3-point scale (0 = “disagree,” 1 = “partly agree,” and 2 = “agree”). Forty items—two of which twice—are condensed into a total score that ranges from 0 to 84. For the current sample, internal consistency was excellent (Cronbach’s α_total_ = 0.94).

The Tinnitus Handicap Inventory (THI [39]; German version [40]) assesses tinnitus-related impairment in daily life activities. It is a self-evaluation instrument and consists of 25 items that are answered on a 3-point scale (0 = “no,” 2 = “occasionally,” and 4 = “yes”). The total score ranges from 0 to 100. For the current sample, internal consistency was excellent (Cronbach’s α_total_ = 0.94).

The Tinnitus Functional Index (TFI [41]; German version [42]) measures negative tinnitus impact. It consists of 25 items that are answered on a 10-point Likert scale with scores ranging from 0 to 100. For the current sample, internal consistency was excellent (Cronbach’s α_total_ = 0.97).

#### 2.6.2. Perceived Stress

Subjective stress was measured using the Perceived Stress Questionnaire (PSQ [43]). The scale consists of 30 items that are rated on a 4-point scale (1 = “almost never”, 2 = “sometimes”, 3 = “often”, and 4 = “almost always”) across three internal (tension, worries, and [lack of] joy) and one perceived external stress dimension (demands). All scores are summed up to a total score for which “joy” is recoded. Each score is linearly transformed to range from 0 to 100. For the current sample, internal consistency was excellent (Cronbach’s α_total_ = 0.91).

#### 2.6.3. Psychological Epiphenomena

The Hospital Anxiety and Depression Scale (HADS) is a screening instrument for self-reported anxiety and depression [44] (German version [45]). The scale features two independent subscales with seven items each that relate to anxious/depressive symptoms during the last week (0 = “not at all” to 3 = “mostly”). For the current sample, internal consistencies were good and excellent, respectively (Cronbach’s α_anxiety_ = 0.85; α_depression_ = 0.90).

The ICD-10-Symptom-Rating (ISR [46]) is a questionnaire that aims to evaluate emotional distress across six subscales (depressive syndrome, anxiety syndrome, obsessive-compulsive syndrome, somatoform disorder syndrome, eating disorder syndrome, and supplementary scale). The ISR consists of 29 items that are answered on a 5-point-scale (0 = “does not apply”, 1 = “hardly applies”, 2 = “somewhat applies”, 3 = “considerably applies”, and 4 = “completely applies”). All item values are averaged to a total score that weighs the supplementary scale score twice. Each score ranges from 0 to 4. For the current sample, internal consistency was excellent (Cronbach’s α_total_ = 0.93).

### 2.7. Statistical Analyses

Sociodemographic information was summarized using descriptive statistics. As expected, given the stratification and randomization protocol, the IIG and DIG did not differ on age, gender, and previous use of hearing aid use at pre-treatment. Step 1: Separate two-way analyses of variance (ANOVA) investigated the effects of Terzo© hearing therapy on [a] tinnitus-related distress, [b] perceived stress, and [c] psychological epiphenomena. We specified “group” [IIG vs. DIG] as between factor, “time” [IIGt_1_-t_2_ vs. DIGwait-t_1_] as within factor and “tinnitus-related distress” [TQ, THI, TFI], “perceived stress” [PSQ], or “psychological epiphenomena” [HADS, ISR] as dependent variables. Cohen’s *d* quantified significant effects where indicated (0.20–0.49 small effect, 0.50–0.80 medium effect, and >0.80 large effect). Step 2: In order to investigate the stability of the identified treatment effects, separate three-tiered repeated-measures ANOVAs were conducted on the significant outcome measures identified in Step 1. For these analyses, data for the IIG and DIG were pooled (*n* = 150). Pooling was possible because computations revealed that [a] the IIG and DIG did not differ on any of the defined outcome variables at their respective pre-treatment timepoint [t_1_], [b] there were no significant “group” [IIG vs. DIG] × “time” interaction effects for any of the outcome measures when considering all three treatment-related measurement timepoints [pre-treatment, post-treatment, and follow-up], and [c] there were no significant covariate effects of previous hearing aid use or hearing aid use during treatment. Where significant, age or hearing ability (PTA) were included as covariates. Post hoc paired-samples *t*-tests investigated between-timepoint differences, given a significant main effect of time. Again, Cohen’s *d* quantified effect sizes. All reported results did not differ for participants with or without previous hearing aid use. See Figure 2 for a visualization of analysis protocols.

## 3. Results

### 3.1. Psychological Variables

Upon inclusion in the study, participants reported (1) low-to-moderate levels of tinnitus-related distress and (2) normal levels of [PSQ-measured] perceived stress, [ISR-measured] anxiety-, depression-, obsession-, somatoform-, and eating-related symptoms, as well as [HADS-measured] anxiety- and depression-related symptoms. By contrast, [ISR-measured] overall psychological epiphenomena levels showed mild elevation.

### 3.2. Effects of Terzo© Hearing Therapy on Tinnitus-Related Distress, Perceived Stress, and Psychological Epiphenomena: Immediate Intervention Group vs. Wait (Delayed Intervention Group)

Participants in the IIG used the hearing aids for 9.33 h/day (*SD* = 4.08). Participants who previously wore hearing aids did not differ in their hearing aid use from the remaining subjects (9.66 [*SD* = 4.06] vs. 9.10 [*SD* = 4.20] hours/day; not significant).

Significant “group” × “time” interaction effects emerged for all three tinnitus questionnaires. Relative to the DIG that yielded stable measurements, the IIG showed significant improvements of tinnitus-related distress (TQ: *F*(1, 158) = 21.21, *p* < 0.001, *d* = −0.304 [small effect]; THI: *F*(1, 158) = 5.02, *p* = 0.026, *d* = −0.137 [negligible effect]); and TFI: *F*(1, 158) = 34.40, *p* < 0.001, *d* = −0.525 [medium effect]). There were no significant interaction effects for perceived stress and psychological epiphenomena; however, significant main effects of time emerged for anxiety and emotional distress (HADS_anxiety_, and ISR_total, anxiety_). See Table 2 for an overview of results.

### 3.3. Stability of Treatment Effects [Pooled Sample]

**Figure 3:**
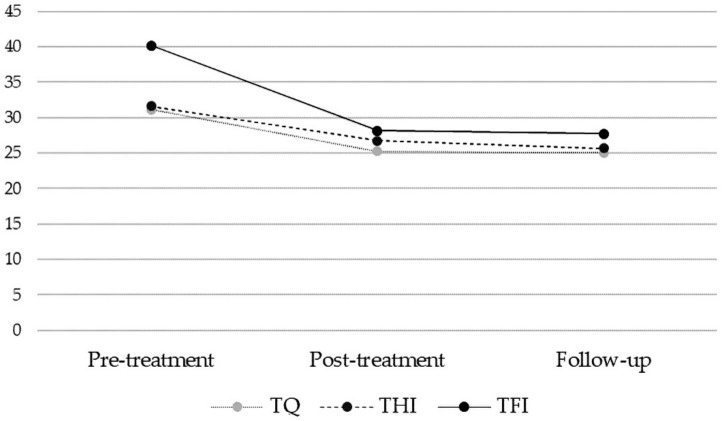
Treatment-related effects on three tinnitus-related distress measures in the pooled total sample (*n* = 150) at pre-treatment, post-treatment, and follow-up. All measures significantly improve from pre- to post-treatment and pre-treatment to follow-up with small (TQ, THI) or medium effect sizes (TFI).

The stability of treatment-related effects was investigated in the pooled dataset (see also ‘2.7. Statistical Analyses’). Terzo-fitted hearing aids were used for 9.49 h/day during the follow-up period (during the intervention period: 9.26 h/day, *SD* = 4.14; *t*(145) = −0.48, not significant). The three-tiered rmANOVAs [pre, post, and follow-up] confirmed significant improvements in tinnitus-related distress (TQ: *F*(1.79, 267.35) = 49.75, *p* < 0.001, *d* = −0.363 [small effect]; THI: *F*(1.73, 257.13) = 23.61, *p* < 0.001, *d* = −0.268 [small effect]; TFI: *F*(1.59, 236.48) = 84.61, *p* < 0.001, *d* = −0.570 [medium effect]). As hypothesized, post hoc comparisons revealed significant improvements from pre- to post-treatment and pre-treatment to follow-up with no significant differences between post-treatment and follow-up. Pre-to-follow-up TQ change comprised 5.8 (*SD* = 8.2), THI change 4.9 (*SD* = 10.9), and TFI 12 (*SD* = 14.6) points. In the context of the overall mild rates of tinnitus-related distress, clinically relevant change (defined as TQ_Follow-up-Pre_ ≥ 12; THI_Follow-up-Pre_ ≥ 7 and TFI_Follow-up-Pre_ ≥ 13) was obtained for 0.7 (TQ), 10.7 (THI), and 2.7% (TFI) of participants, respectively. See Figure 3 for a visualization of results.

### 3.4. Exploratory Analyses: Uncontrolled Effects from Pre-Treatment to Follow-Up [Pooled Sample]

Significant, yet uncontrolled, pre- to follow-up changes emerged for indices of perceived stress (PSQ_total: *F*(1.91, 284.39) = 5.98, *p* = 0.003, *d* = −0.152; PSQ_t: *F*(1.78, 265.62) = 5.07, *p* = 0.009), *d* = −0.165; and PSQ_d: *F*(1.88, 274.30) = 3.70, *p* = 0.029, *d* = −0.122) and emotional distress, notably depressive symptoms and anxiety (HADS_d: *F*(2, 298) = 4.30, *p* = 0.014), *d* = −0.080; HADS_a: *F*(2, 298) = 4.50, *p* = 0.012), *d* = −0.110; ISR_total: *F*(1.89, 278.94) = 3.92, *p* = 0.023, *d* = −0.109; ISR_d: *F*(1.84, 273.56) = 3.90, *p* = 0.025, *d* = −0.115; and ISR_sup: *F*(1.75, 259.29) = 3.79, *p* = 0.029, *d* = −0.100). Whilst statistically significant, the effect sizes for these changes were ‘negligible’ (<0.2). See Table 3 for an overview.

### 3.5. Exploratory Analyses: Hearing Ability and [Pre-to-Follow-Up Change in] Psychological Variables [Pooled Sample]

At pre-treatment [IIG: t_1_; DIG: t_1_], small positive correlations (Pearsons *r* < 0.02) emerged between participants’ hearing ability and emotional distress (*r*_PTA_ISR_Total_ = 0.154, *p* = 0.04), as well as depression (*r*_PTA_ISR_d_ = 0.154, *p* = 0.03). No correlations were found between PTA indices and change across those psychological variables that were found to improve over time.

## 4. Discussion

The present study investigated the effects of Terzo© hearing therapy on tinnitus-related distress and accompanying psychological parameters. The intervention combined binaurally-fitted hearing aids with a 14-day, CD-enhanced Terzo self-study program.

Using a randomized-controlled cross-over design, an immediate (vs. delayed) intervention group showed significantly greater improvements in tinnitus-related distress. This finding demonstrates for the first time that the 14-day Terzo© hearing therapy bears the potential to improve self-reported psychological aspects of tinnitus symptomatology in patients with chronic tinnitus and mild-to-moderate high-frequency hearing loss. Importantly, however, the effects sizes of the observed effects differed according to the respectively used questionnaire (TQ: small effect size; THI: negligible effect size; and TFI: medium effect size) highlighting the importance of matching the content and targets of interventions with respectively applied measurement choices [7].

The study joins previous research on self-reported symptom-relief following hearing aid fittings in patients with hearing loss [19,20,47] with possible mechanisms involving enhancements of individuals’ tonal environments [21,48,49], tinnitus masking [19], reduced tinnitus awareness and improved communication opportunities [22] or possible psychological effects such as, for some patients, enhanced senses of self-efficacy, control, or social inclusion. In the present study, the majority of participants (68%) wore the hearing aids between 5.25 and 13.41 h/day, which is broadly consistent with the previously reported times in the literature (e.g., 3.67–11.93 h/day [50], 5.49–11.77 h/day [51], or 1.20–11.00 h/day [52]).

Because the intervention primarily aimed to improve executive and attention-related processes in their interaction with participants’ hearing ability, it follows that the observed improvements were primarily reflected in the TFI, which, unlike the TQ and THI, emphasizes audiological and functional aspects of chronic tinnitus [7,53].

Whilst the Terzo training was not directly aimed at addressing psychological parameters, we expected indirect psychological benefits, possibly through ameliorating sensory contributions that have been well established in their impact on emotional distress [54]. Contrary to this expectation, we found that emotional distress slightly improved in both groups. By contrast, perceived stress and depressive symptoms did not change in either group. Crucially, upon inclusion in the study, participants showed overall low levels of psychological distress, and the resulting floor effects may limit the possibilities to effect or detect changes with treatment [55,56]. Nonetheless, time-related improvements in anxiety might be attributable to the anticipation of treatment, natural fluctuations in symptoms, coping mechanisms, or self-monitoring skills, as well as - in the immediate intervention group—a possible response–shift bias [57]. Natural improvements in anxiety with time have been previously observed across different patient populations [57,58,59]. However, research on such fluctuations of expectation-based effects has yielded mixed results, and the listed reasons for the observed effects remain speculative at this point. In the present study, we observed no beneficial effect of hearing aids on the indices of depression or perceived stress, which were, however, low upon commencing treatment. Whilst hearing loss has been associated with depression, anxiety, or stress in some studies [25,60,61], but not others [62,63], our findings suggest a small association between mild-to-moderate hearing loss and emotional distress or low mood, albeit within a context of overall low distress and symptom severity. Similarly, evidence for hearing aid-associated improvements in depression is mixed with some studies [64,65], but not others [60,66], reporting hearing aid or auditory-training-related improvements in mood. However, ‘depression’ labels a heterogeneous syndrome cluster that involves interacting cognitive, affective, behavioural, and physiological symptoms [67], and the present study’s findings need to be replicated in study populations with varying levels of low mood - using multidimensional depression measures, that capture experiential as well as physiological aspects of depression-related symptom presentations [68].

Examining treatment effects within the pooled sample, the improvements of tinnitus-related distress remained stable at follow-up. Stable improvements following hearing-focused interventions have been previously reported after cochlear implantation [69,70,71,72] or hearing aid fittings [73], and the present study provides first promising evidence in favour of the here-investigated intervention in this regard.

Finally, exploratory analyses revealed statistically significant, yet negligible, improvements of perceived stress and emotional distress indices between pre-treatment and follow-up that were further unrelated to participants’ hearing ability.

### 4.1. Limitations

Participants in the present study yielded only mild levels of hearing impairment and tinnitus-related, as well as broader psychological distress. Future studies need to investigate the helpfulness of the approach for more severely impaired populations that were excluded in the present study. Moreover, a Terzo employee conducted the hearing aid fittings and the explanatory introduction to the hearing therapy. Whilst this may constitute a source of bias, it is transparently highlighted in the study’s Conflict of Interest section, and all data collection and analysis procedures were conducted independently. Terzo© hearing therapy consists of a combination of hearing aid fitting and auditory training. Future studies might wish to investigate the specific effects of the hearing aids or auditory training components in order to maximize the effects for different subgroups of patients at various levels of distress or (hearing) impairment.

### 4.2. Clinical Implications

The present study offers the first support of the hypothesis that a combination of binaural hearing aid fittings and auditory training (the Terzo© hearing therapy) can improve tinnitus-related distress in patients with chronic tinnitus and mild-to-moderate hearing loss. Whilst methodologically exploratory, the observed benefits of this intervention on psychological indices warrant further investigation, particularly across samples with varying levels of emotional distress.

## Figures and Tables

**Table 1:** Sociodemographic data and patient characteristics (*n* = 177).

		*n*	%
Education			
	Completed junior apprenticeship	72	40.7
	Completed senior apprenticeship	40	22.6
	University degree	60	33.9
	Other	4	2.3
Employment ‘yes’		105	59.3
Relationship status			
	Single	25	14.1
	Married	114	64.4
	Divorced	27	15.3
	Widowed	10	5.6
Duration of tinnitus			
	<0.5 year	5	2.8
	0.5–1 year	9	5.1
	1–2 years	23	13.0
	2–5 years	24	13.6
	>5 years	107	60.5
Tinnitus onset			
	gradual	92	52.0
	sudden	73	41.2
Frequency			
	very high	37	20.9
	high	104	58.8
	middle	32	18.1
	low	3	1.7
Past psychotherapy ‘yes’		53	29.9
Use of hearing aid ‘yes’		53	31.5

**Table 2:** Means, standard deviations, and significant main effects of “time” or “group” × time interaction effects for tinnitus-related distress, perceived stress, and psychological epiphenomena in the immediate vs. delayed intervention groups.

	Group	Timepoint				Group × Time Interaction Effect	*d*	Main Effect of Time	*d*
	*n*_IIG_ = 79 *n*_DIG_ = 81	t_1IIG_; t_wDIG_		t_2IIG_; t_1DIG_					
Measure		*M*	*SD*	*M*	*SD*				
TQ	IIG	31.11	15.72	24.71	15.46	*F*_group × time_(1, 158) = 21.21, *p* < 0.001	−0.304		
	DIG	33.05	17.09	31.67	16.14				
THI	IIG	30.61	22.09	26.20	21.41	*F*_group × time_(1, 158) = 5.02, *p* = 0.026	−0.137		
	DIG	34.17	23.68	32.91	22.06				
TFI	IIG	40.38	21.46	27.85	21.61	*F*_group × time_(1, 158) = 34.40, *p* < 0.001	−0.525		
	DIG	41.87	21.83	40.75	21.68				
PSQ_total	IIG	28.12	19.00	26.60	18.85				
	DIG	30.07	19.59	29.42	19.66				
PSQ_w	IIG	23.46	19.91	20.59	20.18				
	DIG	26.75	23.39	26.58	23.18				
PSQ_t	IIG	34.01	25.41	30.72	23.98				
	DIG	35.12	26.08	34.40	23.70				
PSQ_j	IIG	57.05	27.67	57.62	26.18				
	DIG	57.12	27.37	56.21	28.44				
PSQ_d	IIG	31.98	21.60	31.05	22.39				
	DIG	32.67	22.45	33.00	24.40				
HADS_a	IIG	6.09	4.06	5.30	4.10			*F*_time_(1,158) = 7.98, *p* = 0.005	−0.114
	DIG	6.40	4.55	6.20	4.18				
HADS_d	IIG	5.19	4.44	4.94	4.46				
	DIG	5.73	5.07	5.86	4.91				
ISR_total	IIG	0.59	0.49	0.54	0.52			*F*_time_(1,158) = 8.67, *p =* 0.004	−0.094
	DIG	0.66	0.62	0.61	0.57				
ISR_ds	IIG	0.88	0.95	0.82	0.96				
	DIG	0.98	1.01	0.96	0.97				
ISR_as	IIG	0.90	0.84	0.68	0.73			*F*_time_(1,158) = 13.83, *p* < 0.001	−0.186
	DIG	0.84	0.91	0.75	0.81				
ISR_ocd	IIG	0.49	0.70	0.54	0.85				
	DIG	0.64	0.84	0.58	0.77				
ISR_sds	IIG	0.33	0.51	0.27	0.52				
	DIG	0.48	0.70	0.40	0.67				
ISR_eds	IIG	0.51	0.60	0.54	0.68				
	DIG	0.48	0.73	0.47	0.73				
ISR_sup	IIG	0.52	0.44	0.47	0.42			*F*_time_(1,157) = 9.21, *p =* 0.003	−0.098
	DIG	0.62	0.59	0.58	0.56				

Notes: TQ = Tinnitus Questionnaire—German version; THI = Tinnitus Handicap Inventory; TFI = Tinnitus Functional Index; PSQ = Perceived Stress Questionnaire; PSQ_w = PSQ, worries subscale; PSQ_t = PSQ, tension subscale; PSQ_j = PSQ, joy subscale; PSQ_d = PSQ, demands subscale; HADS_a = Hospital Anxiety and Depression Scale; Anxiety Subscale; HADS_d = HADS, Depression subscale; ISR = IDC-10 Symptom Rating; ISR_ds = ISR, depressive syndrome subscale; ISR_as = ISR, anxiety syndrome subscale; ISR_ocd = ISR, obsessive-compulsive syndrome subscale; ISR_sds = ISR, somatoform disorder syndrome subscale; ISR_eds = ISR, eating disorder syndrome subscale; ISR_sup = ISR, supplementary subscale; IIG = immediate intervention group; DIG = delayed intervention group; *M* = mean; and *SD* = standard deviation. *d* = Cohen’s *d* (0.20–0.49 small effect, 0.50–0.80 medium effect, and > 0.80 large effect).

**Table 3:** Means, standard deviations, and significant pairwise comparisons investigating [a] the stability of identified treatment effects (**t_2-_ t_3_**) and [b] exploratory uncontrolled pre- to follow-up changes in the pooled total sample (**t_1-_ t_3_**).

Group *n* = 150	Timepoint Pre		Post		Follow-Up		Paired Samples *t*-Tests		
	t_1IIG and DIG_		t_2IIG and DIG_		t_3IIG and DIG_		t_2-_ t_3_	t_1-_ t_3_	*d*
Measure	*M*	*SD*	*M*	*SD*	*M*	*SD*			
TQ ^[a]^	31.09	16.16	25.27	15.80	25.07	17.02		*t*(149) = 7.67, *p* < 0.001	−0.363
THI ^[a]^	31.64	22.36	26.75	22.03	25.65	22.42		*t*(149) = 5.50, *p* < 0.001	−0.268
TFI ^[a]^	40.18	21.67	28.14	21.22	27.69	22.16		*t*(149) = 10.02, *p* < 0.001	−0.570
PSQ_total ^[b]^	28.80	19.85	27.12	19.95	25.84	19.16		*t*(149) = 3.13, *p* < 0.01	−0.152
PSQ_w	25.20	22.14	23.16	22.32	23.24	22.73			
PSQ_t ^[b]^	34.31	25.16	30.80	23.79	30.18	25.01		*t*(149) = 2.60, *p* < 0.05	−0.165
PSQ_j ^2[b]^	57.20	28.49	59.19	28.49	60.31	28.29			
PSQ_d ^1[b]^	32.36	23.36	31.29	23.58	29.56	21.97		*t*(149) = 2.21, *p* < 0.05	−0.122
HADS_a ^[b]^	6.04	4.19	5.58	4.42	5.55	4.69		*t*(149) = 2.35, *p* < 0.05	−0.110
HADS_d ^[b]^	5.47	4.78	5.02	4.85	5.09	4.71		*t*(149) = 2.15, *p* < 0.05	−0.080
ISR_total ^2[b]^	0.60	0.54	0.56	0.56	0.54	0.56		*t*(149) = 2.79, *p* < 0.01	−0.109
ISR_ds ^[b]^	0.93	0.97	0.87	0.98	0.82	0.94		*t*(149) = 2.45, *p* < 0.05	−0.115
ISR_as ^2[b]^	0.83	0.84	0.67	0.75	0.69	0.82			
ISR_ocd	0.51	0.73	0.53	0.81	0.49	0.75			
ISR_sds	0.35	0.58	0.34	0.58	0.30	0.58			
ISR_eds	0.48	0.68	0.47	0.71	0.48	0.74			
ISR_sup ^2[b]^	0.55	0.52	0.51	0.54	0.50	0.53		*t*(149) = 2.62, *p* < 0.05	−0.100

Notes: TQ = Tinnitus Questionnaire—German version; THI = Tinnitus Handicap Inventory; TFI = Tinnitus Functional Index; PSQ = Perceived Stress Questionnaire; PSQ_w = PSQ, worries subscale; PSQ_t = PSQ, tension subscale; PSQ_j = PSQ, joy subscale; PSQ_d = PSQ, demands subscale; HADS_a = Hospital Anxiety and Depression Scale; Anxiety subscale; HADS_d = HADS, Depression subscale; ISR = IDC-10 Symptom Rating; ISR_ds = ISR, depressive syndrome subscale; ISR_as = ISR, anxiety syndrome subscale; ISR_ocd = ISR, obsessive–compulsive syndrome subscale; ISR_sds = ISR, somatoform syndrome subscale; ISR_eds = ISR, eating disorder syndrome subscale; ISR_sup = ISR, supplementary subscale; *M* = mean; *SD* = standard deviation. *d* = Cohen’s *d* (0.20–0.49 small effect, 0.50–0.80 medium effect, and > 0.80 large effect). ^[a]^ Identified controlled treatment effects and ^[b]^ exploratory uncontrolled pre- to follow-up changes. ^1^ Significant covariate “age“ included in model. ^2^ Significant covariate “mean pure tone audiometry” included in model.

## Data Availability

As per Charité Universitätsmedizin Berlin’s ethics committee, unfortunately, we cannot make the data public without restrictions because we did not obtain patients’ consent to do so at the time. Nevertheless, interested researchers can contact the directorate of the Tinnitus Center Charité Universitaetsmedizin Berlin with data access requests (birgit.mazurek@charite.de).
